# Anti-Photoaging Effects of Nanocomposites of Amphiphilic Chitosan/18β-Glycyrrhetinic Acid

**DOI:** 10.3390/molecules28114362

**Published:** 2023-05-26

**Authors:** Weiyan Quan, Songzhi Kong, Sidong Li, Qianqian Ouyang, Sitong Lu, Jiaqi Guo, Kefeng Wu, Wei Zhao, Hui Luo

**Affiliations:** 1 Marine Biomedical Research Institute, Guangdong Medical University, Zhanjiang 524023, China; quanfei183@126.com (W.Q.);; 2 Department of Applied Chemistry, School of Chemistry and Environmental Science, Guangdong Ocean University, Zhanjiang 524088, China; 13702737491@163.com (S.L.);; 3 Songshan Lake Materials Laboratory, Dongguan 523808, China; 4 Guangdong Provincial Key Laboratory of Tropical Disease Research, School of Public Health, Southern Medical University, Guangzhou 510515, China

**Keywords:** amphiphilic chitosan, 18β-glycyrrhetinic acid, nanocrystals, hydrogel, skin photoaging

## Abstract

Improving the transdermal absorption of weakly soluble drugs for topical use can help to prevent and treat skin photoaging. Nanocrystals of 18β-glycyrrhetinic acid (i.e., NGAs) prepared by high-pressure homogenization and amphiphilic chitosan (ACS) were used to form ANGA composites by electrostatic adsorption, and the optimal ratio of NGA to ACS was 10:1. Dynamic light scattering analysis and zeta potential analysis were used to evaluate the nanocomposites’ suspension, and the results showed that mean particle size was 318.8 ± 5.4 nm and the zeta potential was 30.88 ± 1.4 mV after autoclaving (121 °C, 30 min). The results of CCK-8 showed that the half-maximal inhibitory concentration (IC50) of ANGAs (71.9 μg/mL) was higher than that of NGAs (51.6 μg/mL), indicating that the cytotoxicity of ANGAs was weaker than that of NGAs at 24 h. After the composite had been prepared as a hydrogel, the vertical diffusion (Franz) cells were used to investigate skin permeability in vitro, and it was shown that the cumulative permeability of the ANGA hydrogel increased from 56.5 ± 1.4% to 75.3 ± 1.8%. The efficacy of the ANGA hydrogel against skin photoaging was studied by constructing a photoaging animal model under ultraviolet (UV) irradiation and staining. The ANGA hydrogel improved the photoaging characteristics of UV-induced mouse skin significantly, improved structural changes (e.g., breakage and clumping of collagen and elastic fibers in the dermis) significantly, and improved skin elasticity, while it inhibited the abnormal expression of matrix metalloproteinase (MMP)-1 and MMP-3 significantly, thereby reducing the damage caused by UV irradiation to the collagen-fiber structure. These results indicated that the NGAs could enhance the local penetration of GA into the skin and significantly improve the photoaging of mouse skin. The ANGA hydrogel could be used to counteract skin photoaging.

## 1. Introduction

The photoaging of skin seriously affects physical appearance but is also linked to skin cancer [1]. Therefore, exploring natural and highly effective substances that could be employed to prevent and/or treat skin photoaging is a rational approach.

Many natural components of plant origin (e.g., Ramulus Mori extract, licorice extract, liquiritin, vitamin C) have shown robust antioxidant properties and anti-aging effects on the skin [2]. Licorice extract (18β-glycyrrhetinic acid (GA)) not only has potent antioxidant properties but also has excellent anti-inflammatory activity [3,4], is applied in intestinal disorders [5], and has a significant effect on resisting ultraviolet (UV)-light-induced damage to the skin [6]. However, GA is insoluble in water and is not absorbed readily and utilized, which affects its curative effect. To overcome the shortcomings of GA delivery, Jin et al. [7] designed reactive oxygen species–responsive polymer–drug conjugate nanoparticles to improve the efficacy of cerebral ischemia therapy, and it presented excellent therapeutic efficacy in stroke mice. Even so, improving the bioavailability of GA in dermal and transdermal drug delivery is a challenge.

Nanocrystal technology is one of the best ways to address the bioavailability of insoluble drugs [8]. Nanocrystalline drugs are carrier-free, submicron, colloidal-dispersion systems in which the drug substance is micronized directly to the nanoscale. In general, the particle size is 100–1000 nm, and this dispersion system contains only the drug and a stabilizer, which act to reduce the aggregation of drug crystals and improve the stability of the product [9]. Nanocrystalline formulations are used widely in drug delivery because of high drug loading and greatly improved druggability, especially for class-II and -IV drugs in biopharmaceutical classification systems. Nanocrystalline formulations can be administered through the gastrointestinal tract for cancer treatment [10] and topically for the treatment of skin disorders [11], thereby representing a novel approach to overcome the skin barrier [12]. Quan and colleagues found that GA, as a fat-soluble drug, could penetrate the skin more deeply after being nanosized [13]. It has been reported that coating camptothecin nanocrystals with dopamine can improve the bioavailability of camptothecin and achieve more efficacious anticancer effects [14]. Therefore, we postulated that coating nanocrystals of 18β-glycyrrhetinic acid (i.e., NGAs) with amphiphilic chitosan (ACS) could promote the permeability of GA, improve its bioavailability, and achieve an efficacious anti-photoaging effect.

Chitosan is the only alkaline polysaccharide in nature. Compared with many other natural polymers, chitosan is used widely in drug-delivery materials, gene-transfer carriers, absorbable materials used in medicine, and for drug targeting because of its nontoxic, nonantigenic, positively charged molecules, as well as good bioadhesive and biodegradable properties [15,16,17,18]. However, chitosan is almost insoluble in water due to its strong intramolecular hydrogen bonds, which limits its application. Therefore, the modification of chitosan could improve its range of applications [19]. In view of the hydrophilic and hydrophobic groups of ACS, micelles with a core–shell structure can be self-assembled through intramolecular and intermolecular interactions for drug delivery [20]. In addition, ACS has some unreacted amino groups with positive charges, which can improve the drug capture of cells through electrostatic adsorption with the negatively charged cell surface [21].

Previously, we prepared ACS with a hydrophilic group (sialic acid) and a hydrophobic group (GA) and showed that the as-prepared ACS derivatives were positively charged in water and had good biocompatibility, indicating that ACS derivatives could be used in the preparation of biologic agents [22]. We also prepared NGAs using a high-pressure microjet method and explored their morphology and their zeta potential, permeation properties, solubility, and anti-inflammatory effects. We showed that, after nanosizing the GA, although the surface of the NGAs was negatively charged, the permeation properties were enhanced, and the solubility and the anti-inflammatory effect improved significantly [13]. In addition, GA has shown a good anti-photoaging effect [6] but low bioavailability (resulting from GA being almost insoluble in water) and weak penetration of skin. Therefore, combined with our previous findings, ACS derivatives with positive charges were blended with negatively charged NGAs via electrostatic adsorption to achieve a more potent photoaging effect of GA.

## 2. Results

### 2.1. Preparation of ANGA Composites

The ANGA composites were prepared by simple blending. The most suitable proportion was obtained by measuring the zeta potential and mean particle size (MPS) of the composite formed in different proportions (Table 1). If ACS was not added, the zeta potential of the NGA suspension was −36.8 ± 0.70 mV. Upon the addition of ACS, the zeta potential of the composite increased. When the ACS:NGA ratio was 1:10, the zeta potential of the composite increased to 31.63 ± 0.76 mV, which just exceeded the minimum zeta potential (30 mV) at which a stable dispersion system could be achieved. The MPS of the composite increased gradually with an increase in the amount of ACS compared to that when no ACS was present at the start (290.6 ± 7.3 nm). When the ACS:NGA ratio was 1:10, the MPS reached 314.3 ± 4.5 nm. Then, with a continuous increase in the amount of ACS added, the MPS increased only slightly. In addition, the polydispersity index (PDI) of each system was low, indicating that the particle-size distribution was narrow. With an increase in ACS content, the composite changed from a negatively charged stable system to a positively charged stable system. The MPS of the composite also increased with an increase in ACS content and reached adsorption saturation gradually. Considering the zeta potential and MPS, the optimal ACS:NGA ratio was 1:10.

**Figure 1:**
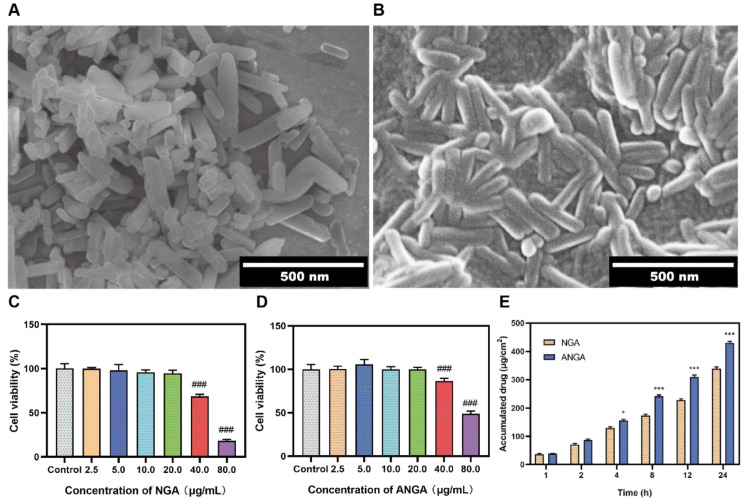
Scanning electron micrographs of NGAs (**A**) and ANGAs (**B**), effects of different concentrations of NGAs (**C**) and ANGAs (**D**) on the survival of HaCaT cells, cumulative amount permeating (**E**) through mouse skin layers 24 h after application of the NGA hydrogel or ANGA hydrogel (mean ± SD, *n* = 3). Control group vs. concentration of 40.0 group, 80.0 group, respectively, ### *p* < 0.005, NGAs vs. ANGAs, * *p* < 0.05, *** *p* < 0.005.

The morphology of the NGAs and ANGAs were visualized by SEM. Each NGA was a rectangular crystal with clear prisms (Figure 1A). Upon the addition of ACS, the morphology of each NGA changed to a well-rounded cylinder (Figure 1B). The ACS formed a complex with the NGAs.

### 2.2. Effects of Sterilization on the MPS and Zeta Potential

We examined changes in the MPS and zeta potential between an NGA suspension and the ANGA suspension before and after sterilization (121 °C, 30 min) (Table 2). The MPS of the NGA suspension increased from 290 nm to 360 nm after sterilization, whereas that of the ANGA suspension did not change significantly, which may have been because ACS was a sterically stable and sequestering agent that prevented the NGAs from aggregating. The zeta potential and PDI of the NGA suspension and the ANGA suspension did not change significantly after autoclaving.

### 2.3. Cytotoxicity of ANGA Composites

Figure 1C,D show the effects of different concentrations of NGAs and ANGAs on the survival of HaCaT cells after 24 h of culture. Compared with the control group, after 24 h of treatment, the NGAs (Figure 1C) and ANGAs (Figure 1D) at low concentrations (2.5–20.0 μg/mL) did not inhibit the proliferation of HaCaT cells significantly; at high concentrations (40.0–80.0 μg/mL), the proliferation of HaCaT cells was inhibited significantly. Hence, the NGAs and ANGAs were cytotoxic at high concentrations. The half-maximal inhibitory concentration (IC_50_) of ANGAs (71.9 μg/mL) was higher than that of NGAs (51.6 μg/mL), indicating that the cytotoxicity of ANGAs was weaker than that of NGAs at 24 h. Furthermore, at concentrations of 40.0 and 80.0 μg/mL, the mean viability of ANGA-treated cells was 86.3% and 48.8%, respectively, which was significantly higher than that of NGA-treated cells (68.6% and 18.3%, respectively).

### 2.4. Skin Penetration of the ANGA Hydrogel In Vitro

The skin-penetration properties of the NGA hydrogel and the ANGA hydrogel in vitro were investigated using Franz cells. Table 3 lists the percent drug retention and cumulative permeability after 24 h. The difference in percent drug retention of the NGA hydrogel and ANGA hydrogel in the skin was negligible, which may have been due to a large amount of drug entering the skin after 24 h of penetration, resulting in saturation of the drug concentration in the skin. However, the skin permeability of the ANGA hydrogel and NGA hydrogel differed greatly, increasing from 56.5% to 75.3% for the ANGA hydrogel.

Figure 1E shows the cumulative penetration of the NGA hydrogel and the ANGA hydrogel through the epidermis at 1, 2, 4, 8, 12, and 24 h. After 2 h, the cumulative penetration of the ANGA hydrogel was significantly higher than that of the NGA hydrogel (*p* < 0.05, *p* < 0.005). After 12 h, the ANGA hydrogel continued to maintain strong permeability, and the cumulative permeability reached 430 μg/cm^2^ at 24 h, whereas that of the NGA hydrogel was 339 μg/cm^2^. These results showed that the skin-penetration ability of the ANGA hydrogel was greater than that of the NGA hydrogel.

### 2.5. ANGA Hydrogel Ameliorated the Macroscopic Appearance of Photoaged Mouse Skin

**Figure 2:**
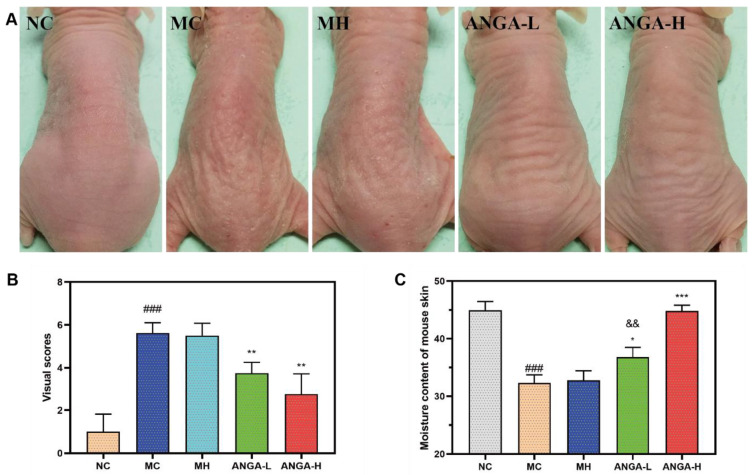
Protective effect of the ANGA hydrogel on the back skin of UV-exposed mice. Macroscopic image of the back skin of each group of mice (**A**), visual score (**B**), and moisture content of mouse skin (**C**) (mean ± SD, *n* = 8). NC vs. MC, ### *p* < 0.005; MC vs. ANGA-L, ANGA-H, respectively, * *p* < 0.05, ** *p* < 0.01, *** *p* < 0.005, ANGA-L vs. ANGA-H, && *p* < 0.01.

The macroscopic effect of UV on mouse skin is shown proved in Figure 2. The dorsal skin of the NC-group mice was smooth with only a few small wrinkles. After 10 weeks of UV irradiation, the skin of the MC-group mice showed erythema, dryness, thickening, sagging, coarse wrinkles, leathery appearance, and mild lesions (Figure 2A), and the visual score was significantly higher than that of the NC-group mice (Figure 2B). These results showed that repeated UV irradiation could cause photodamage to the dorsal skin of mice. There was no significant difference in the macroscopic characteristics of skin between mice in the MH group and the MC group, indicating that the matrix of the ANGA hydrogel had no significant effect on skin photoaging. However, these UV-induced skin changes were restored (at least in part) by topical treatment with the ANGA hydrogel. Especially when the ANGA hydrogel was at a high concentration (ANGA-H), the dorsal skin of mice exhibited smoothness and shallow wrinkles. Consistent with the observations made above, topical application of the ANGA hydrogel decreased the skin score significantly (Figure 2A,B). This finding indicated that topical application of the ANGA hydrogel after sustained UV irradiation for 10 weeks had an inhibitory effect on macroscopic skin damage in mice.

Long-term UV irradiation reduced the moisture content of mouse skin, resulting in accelerated skin aging (Figure 2C). Compared with the MC-group mice, the skin moisture content of the NC-group mice decreased from 44.9% to 32.3%, indicating that UV irradiation had a significant effect on the skin moisture of mice. After treatment with the ANGA hydrogel, compared with the MC group, the water content of each group increased significantly. Among them, the moisture content of the ANGA-H group reached 44.8%, which was close to that of the NC group (44.9%). Hence, the ANGA hydrogel could increase the water content in the skin tissue of UV-induced photoaged mice significantly.

### 2.6. ANGA Hydrogel Prevented UV-Induced Damage to Skin Structure

**Figure 3:**
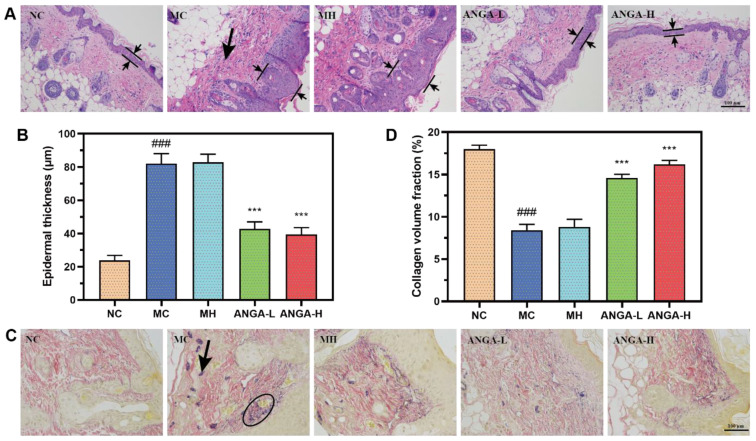
Histological changes of skin in UV-exposed mice (200× magnification) (**A**). Skin epidermal thickness of UV-exposed mice (mean ± SD, *n* = 8) (**B**). Effect of the ANGA hydrogel on the elastic fibers in skin tissue of UV-exposed mice (200× magnification) (**C**). Collagen content of mouse skin tissue (mean ± SD, *n* = 8) (**D**). NC vs. MC, ### *p* < 0.005; MC vs. ANGA-L, ANGA-H, respectively, *** *p* < 0.005.

Compared with the NC group, the MC group showed increased epidermal thickness, dermal-layer thinning, inflammatory-cell infiltration, and a relative reduction in collagen content (H&E staining; Figure 3A). After treatment with the ANGA hydrogel, the skin condition of mice in the low-dose group and the high-dose group was alleviated to a greater extent than that of the MC-group mice. That is, the epidermal layer was significantly thinner and sharply demarcated from the dermal layer, and inflammatory-cell infiltration in the dermal layer was alleviated; the high-dose group showed greater improvement than the low-dose group. These results indicated that the ANGA hydrogel could slow down the inflammation of skin tissues in photoaged mice.

Irregular thickening of the epidermal layer (i.e., epidermal hyperplasia) is often used as a parameter to evaluate inflammation and wrinkle formation in skin photoaging [23]. Hence, we measured the mean thickness of the epidermis of the mouse skin in each group with the aid of H&E staining of skin samples (Figure 3B). After UV irradiation, the skin of the MC-group mice was significantly thicker than that of the NC-group mice. After treatment with the ANGA hydrogel, low-dose and high-dose groups could slow down the increased skin thickness of photoaged mice. This finding indicated that the ANGA hydrogel could inhibit inflammation in photoaged mouse skin significantly.

### 2.7. ANGA Hydrogel Protected the Integrity of the Fiber Structure

Elastic fibers are important parts of the skin structure, and key components for preserving skin elasticity. We used staining (resorcin-fuchsin) to contrast the state of elastic fibers on the dorsal skin of the mice in each group. We studied the protective effect of the ANGA hydrogel on elastic fibers on the dorsal skin of photoaged mice (Figure 3C). The elastic fibers of the NC-group mice (stained blue) were elongated and wavy in an orderly arrangement and distributed evenly around collagen fibers. In the MC group, elastic fibers were thickened and twisted, partly coiled in clumps, and distributed unevenly (arrow and circle in Figure 3C). Treatment of mice with low and high doses of the ANGA hydrogel led to a significant improvement in the structural morphology and alignment distribution of elastic fibers. The structure of elastic fibers in the skin of mice in the high-dose group was restored to a network-like, orderly arrangement, and occasionally, aggregate entanglement was seen. Overall, the elastic fibers were more robust in the high-dose group than in the low-dose group. This finding suggested that the ANGA hydrogel could slow down the UV-induced damage to elastic fibers and maintain their normal morphology and distribution.

**Figure 4:**
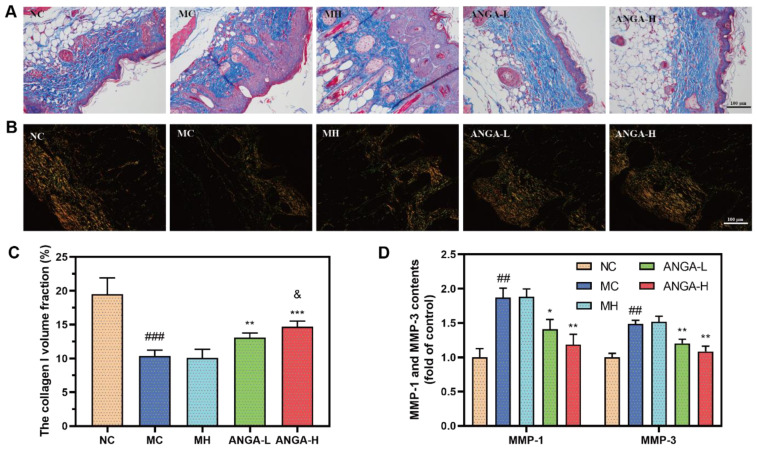
Masson’s trichrome staining of mouse skin tissue (200× magnification) (**A**). Sirius Red staining of mouse skin tissue (200× magnification) (**B**). Content of collagen I in mouse skin tissue (mean ± SD, *n* = 8) (**C**). Effects of the ANGA hydrogel on expression of MMP-1 and MMP-3 in photoaged mouse skin tissue (mean ± SD, *n* = 8) (**D**). NC vs. MC, ## *p* < 0.01, ### *p* < 0.005; MC vs. ANGA-L, ANGA-H, respectively, * *p* < 0.05, ** *p* < 0.01, *** *p* < 0.00; ANGA-L vs. ANGA-H, & *p* < 0.05.

Staining with Masson’s trichrome was used to examine the distribution and morphology of collagen fibers in the dermis of mice (Figure 4A). The collagen fibers of mice in the NC group were bundle-like, arranged in an orderly fashion, and distributed evenly in the dermal layer. Collagen fibrils in the dermis of the MC-group mice decreased in content in response to epidermal keratinized thickening and inflammation and showed flocculent, unevenly distributed photoaged features. After treatment with the ANGA hydrogel, the distribution and morphology of collagen fibrils in the dermis of the mice were improved significantly, and the collagen content increased significantly (Figure 3D). These results indicated that the ANGA hydrogel could alleviate damage to collagen fibers in the dermis of photoaged mouse skin.

Sirius red staining was employed to examine the distribution and morphology of type-I collagen fibers in the dermis of the photoaged mice [24]. Image-Pro™ Plus 6.0 (Media Cybernetics, San Diego, CA, USA) was used to evaluate the content of type-I collagen within the dermal tissues (Figure 4B,C). The type-I collagen fibers, with strong refractoriness and bright-yellow color in the dermis of mice, in the NC group appeared thick and closely aligned and were entangled by a small amount of type-III collagen with weak refractoriness and greenish color. Type-I collagen fiber bundles in the dermis of the MC-group mice were distorted, fractured, and aggregated abnormally, and their relative content was significantly lower than that of the NC-group mice. Local administration of the ANGA hydrogel could alleviate the UV-induced structural degeneration of type-I collagen fibers and increase the content of type-I collagen; the content of type-I collagen fibers in the high-dose group was higher than that in the low-dose group. These results indicated that the ANGA hydrogel could increase the content of type-I collagen in the skin of photoaged mice significantly.

### 2.8. ANGA Hydrogel Reversed the UV-Induced Increase in MMP Content

Studies have shown that MMP overexpression in skin tissue is a major contributor to photoaging [25]. Hence, ELISAs were employed to examine the effects of the ANGA hydrogel on MMP expression in the skin tissues of photoaged mice (Figure 4D). Compared with the NC group, the expression of MMP-1 and MMP-3 in the MC-group tissues was increased. After treatment with the ANGA hydrogel, expression of MMP-1 and MMP-3 in tissues was reduced significantly. These results indicated that the ANGA hydrogel had an inhibitory effect on expression of MMP-1 and MMP-3 in UV-induced mouse skin tissues.

## 3. Discussion

GA, one of the components in licorice, has adrenocorticotropic-hormone-like effects and exhibits robust anti-inflammatory effects [26]. However, the application of GA is limited by its low solubility in water, which leads to its poor bioavailability after administration. Numerous studies have shown that nanocrystals are ideal for enhancing drug bioavailability [27,28]. Previously, we showed that after GA had been prepared as nanocrystals [13], the solubility of the drug was improved and the cumulative amount permeating through the skin increased; thereby, more drugs were involved in the metabolic process.

To further improve the permeability of NGAs in the stratum corneum, we prepared ACS-encapsulated NGAs (i.e., ANGAs). We took advantage of the fact that the surface of NGAs is negatively charged and ACS is positively charged. The ANGA hydrogel had greater skin permeability than the NGA hydrogel, which indicated that the ANGA hydrogel had potential applications in anti-skin photoaging.

Research on photoaging is reliant on animal models. Hairless mice are used to avoid the tedious process of repeated hair removal and to facilitate chronic photodamage during modeling [29,30,31]. In the present study, BALB/c-nu/nu mice were irradiated with simulated normal sunlight as a photoaging model. After 10 weeks of irradiation, the mice showed obvious photoaging characteristics as well as significantly lower skin moisture. Hence, a photoaging model was successfully established.

Numerous studies have shown that skin is exposed to UV light chronically, resulting in tissue changes, such as elastic-fiber degeneration in the dermal layer and reduced content of collagen fibers. Elastic fibers are key components in keeping the skin smooth and elastic. Collagen fibers maintain skin toughness and filling and prevent the development of wrinkles, which are a direct cause of changes in skin appearance [32,33]. In our study, collagen and elastic fibers were studied by staining. After treatment with the ANGA hydrogel, the pathologic changes in the skin tissue were improved significantly: significant inhibition of inflammation, reduction in epidermal thickness and elastic-fiber clusters, and a significant increase in type-I collagen content.

However, UV does not disrupt extracellular matrix (ECM) components (e.g., collagen fibrils) directly. Studies have shown that MMPs are one of the initiating agents in the degradation of collagen fibrils [34]. Most matrices outside a cell can be hydrolyzed, which is one of the root causes of photoaging [25,35]. MMP-1 degrades various types of collagen fibrils, including type-I and -III. MMP-3 can degrade multiple types of collagen fibrils (e.g., type-III and -V) but can also bind with MMP-1 to degrade most of the ECM in the dermis, thereby rendering the skin wrinkled, loose, rough, and without features such as elasticity. We measured the expression of MMP-1 and MMP-3 using ELISAs. UV irradiation induced significant expression of MMP-1 and MMP-3, and the ANGA hydrogel inhibited the expression of MMP-1 and MMP-3 significantly, with the high-dose group showing greater inhibition than the low-dose group.

## 4. Materials and Methods

### 4.1. Ethical Approval of the Study Protocol

Animal handling/care procedures were undertaken in compliance with the relevant guidelines and regulations set by the National Institutes of Health for the Care and Use of Laboratory Animal and were approved by the Animal Research Ethics Committee of Guangdong Medical University (Laboratory Animal Use Permit Number: SYXK (Yue) 2019-0204) in Zhanjiang, China.

### 4.2. Materials

GA (purity > 98%) was purchased from Nanjing Dilger Medical Technology (Nanjing, China). Sialic acid–chitosan–18β-glycyrrhetinic acid (ACS) conjugates were made by our research team. HaCaT cells were supplied by Guangzhou University of Chinese Medicine (Guangzhou, China). The staining solutions (hematoxylin and eosin (H&E), Masson’s trichrome, Sirius red, and resorcin-fuchsin) were purchased from Wuhan Servicebio Technology (Wuhan, China). Glycerol, F188, and all other reagents were sourced from Macklin Biochemicals (Shanghai, China).

### 4.3. Preparation of NGA and ANGA Suspension

**Scheme 1:**
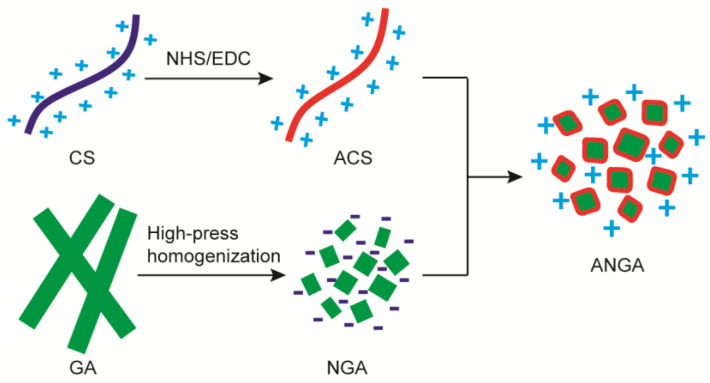
Preparation of ANGA nanocomposites.

ACS and the suspension of stabilized NGAs were prepared according to our previous methods with appropriate modifications (Scheme 1) [13,22]. ACS was synthesized by means of the EDC/NHS coupling chemical reaction. An NGA nanosuspension was obtained by high-pressure homogenization. For the NGA nanosuspension, GA (1.00 g) was dispersed in an aqueous solution of F188 (0.0675%, *w*/*v*, 500.00 mL) with magnetic stirring (1000 rpm, 10 min, room temperature). F188, which is a nonionic and hydrophilic polymer, was used as stabilizer for the dispersion of NGAs. It was homogenized at 20,000 rpm for 1 min using a high-speed dispersion machine (Ultra Turrax T18; IKA, Staufen, Germany). Then, the suspension was transferred to a high-pressure homogenizer (AMF-5; ATS Engineering, Shuzhou, China) working at 1500 bar to prepare the NGA nanosuspension with a concentration of GA of 2 mg/mL.

To obtained the ANGA nanosuspension, an ACS solution in acetic acid was prepared at various concentrations (0.1, 0.2, 0.4, 2.0 mg/mL), and then, 10 mL of ACS solution (pH = 5.0) was added to the NGA nanosuspension (10 mL, 2 mg/mL) with magnetic stirring for 1 h at room temperature to obtain a mass ratio of ACS:NGA of 1:20, 1:10, 1:5, and 1:1, respectively.

The suspension of NGAs and ANGAs was prepared for different concentrations (2.5, 5.0, 10.0, 20.0, 40.0, 80.0 μg/mL) and then placed in an autoclave and sterilized at 121 °C for 30 min for cell viability assay use.

### 4.4. Scanning Electron Microscopy (SEM)

The shape and ACS coating of the fabricated NGA nanoparticles were confirmed by SEM employing a S4800 system (Hitachi, Tokyo, Japan). To accomplish this, fabricated nanoparticles (ACS:NGA = 1:10) were completely dried in a vacuum and then fixed on conductive tape. The sample was coated with gold for 2 min using a sputter coater (DSCR; Vac Coat, London, UK) and observed under an excitation voltage of 5 kV.

### 4.5. Cell Viability Assay

The cytotoxicity of suspensions of NGAs and ANGAs to HaCaT cells was assessed using the Cell Counting Kit 8 (CCK-8) assay. The cell suspension (100 μL) was transferred to a 96-well plate at 8 × 10^3^ cells/mL. Then, the 96-well plate was placed in a cell incubator. After 24 h, the medium was removed, and the cells were washed with phosphate-buffered saline (PBS). Then, we added 100 μL of the NGA suspension or ANGA suspension at different concentrations. After incubation for 24 h, CCK-8 solution (10 μL) was added to each well, followed by incubation for 2 h. The absorbance was measured at 450 nm using a microplate reader (Epoch; Bio Tech, Winooski, VT, USA), and the cell viability was calculated.

### 4.6. Preparation of an ANGA Hydrogel

The lyophilized powder of ANGAs (0.86 g, ACS:NGA = 1:10) with 70% drug loading was added to distilled water (9.14 mL) and stirred well, and 20.0 g of glycerol was added to it. Then, a high-dose ANGA (H-ANGA) hydrogel (GA = 20.0 mg/g) was obtained after thorough stirring. A low-dose ANGA hydrogel (L-ANGA) with a GA content of 5 mg/g and a blank matrix hydrogel (MH) were obtained by the same method.

### 4.7. Skin Permeation Ex Vivo

Male ICR mice (6 weeks) were purchased from the Guangdong Medical Experimental Animal Center (Production Certificate Number: SCXK (Yue) 2019-0035) in Guangdong, China. The mice were killed by cervical dislocation, and their fur was shaved off. Skin from the dorsal region was excised and mounted on vertical diffusion (Franz) cells (RYJ-6B; Huanghai, Shanghai, China) between the donor compartment and receptor compartment, with the stratum-corneum side facing the donor compartment. The donor medium comprised 0.16 g of hydrogel containing GA. The receptor compartment was filled with 6.90 mL of physiologic (0.9%, *w*/*v*) saline, stirred continuously at 500 rpm, at 37 ± 0.1 °C. The permeation area between compartments was 2.8 cm^2^. After 1, 2, 4, 8, 12, and 24 h, aliquots of the receptor medium (0.20 mL) were sampled and replaced immediately with equal volumes of fresh 0.9% saline. The cumulative amounts of the drugs were quantified by high-performance liquid chromatography (HPLC). After 24 h, the skin specimens were washed gently, and the drug content in the supernatant after homogenization and centrifugation was determined by HPLC (Waters Corp., Milford, MA, USA). Separation was performed on a C18 column (4.6 mm × 250 mm; 5 μm; Dikma Technologies, Beijing, China); methanol and water (90:10 *v/v*) were used as the mobile phase at a flow rate of 1.0 mL/min. The analytes were detected by a UV detector at 250 nm.

### 4.8. Preparation of a Photoaged Mouse Model

Ultraviolet-A (UVA) at a power of 13.6 W, UVB at a power of 3 W, and a UV-simulating solar bulb at a total power of 300 W (Osram, Munich, Germany) were employed. They were mounted on top of a homemade simulated daylight irradiator to sunbathe the mice. The mice in each group were moved individually inside a homemade wire-braided cage (~3 cm in height). This cage was moved horizontally into a homemade daylight simulator on a horizontal surface ~30 cm from the UV lights, followed by irradiation. The UV lamp was preheated for 10 min before being used. According to our previous study [5], irradiation was undertaken for 6 days per week. The duration of irradiation was 45 s for 3 days at the beginning of week-1, 1 min for 3 days at the end of week-1, 2 min every day of week-2, 3 min every day of week-3, and 4 min every day of week-4, but the irradiation duration from week-5 to week-10 was not increased. Throughout the experimental period, the mice’s skin was observed closely before and after irradiation each day for erythema, blisters, erosions, and other phenomena. If any of these conditions were visible, irradiation was stopped immediately for 2–3 days, and the experiment continued only after the symptoms had disappeared.

### 4.9. Grouping of Experimental Animals and GA Treatment

BALB/c-nu/nu mice were purchased from the Guangdong Medical Experimental Animal Center (Production Certificate Number: SCXK (Yue) 2019-0035) in Guangdong, China. There were 80 BALB/c-nu/nu mice divided randomly into 5 groups of 16 each: normal (NC), model (MC), matrix control (MH), ANGA low-dose (ANGA-L, 1 mg/g), and ANGA high-dose (ANGA-H, 4 mg/g). In each group, eight mice were used for the measurement of skin content, total protein content, and preparation of tissue sections; the other eight mice were used for the measurement of biochemical indices. Eight mice were housed in each cage. The mice had free access to chow and water under identical environmental conditions. Before experimentation, the mice were allowed to acclimatize to their new environment.

In the NC group, the mice had free access to food and water throughout the experimental period, without any other treatment. In all other groups, from week-1 to week-10, the mice underwent UV irradiation six times per week (no irradiation on Sunday). In the MC group, the mice underwent UV irradiation only. In the MH group, after irradiation, the matrix hydrogel was applied to the back skin at 0.2 g/mouse. In the ANGA-L group, after irradiation, a low dose of ANGA hydrogel was applied to the back skin at 0.2 g/mouse. In the ANGA-H group, after irradiation, a high dose of ANGA hydrogel was applied to the back skin at 0.2 g/mouse.

### 4.10. Macroscopic Evaluation of Dorsal Skin

Referring to previous work [6], at week-10, the mice were anesthetized with ether, and the skin in the middle of the back was photographed. An observer who had clinical experience with skin photoaging and was unaware of the experimental protocol was invited to observe the macroscopic characterization of the back skin of the mice and give the corresponding aging score for each mouse (Table 4).

### 4.11. Determination of Skin Moisture Content

The mice were killed by cervical dislocation (eight mice/group). The full-thickness skin at the experimental site on the back was removed rapidly. The connective tissue and subcutaneous fat were peeled off. About 0.2 g of the skin sample was cut promptly and weighed accurately with an analytical balance. Subsequently, it was placed in an oven to dry at 80 °C to reach a constant weight. Then, the skin sample was removed and weighed again with the analytical balance. The water content of the skin sample was calculated from the weight change of the sample before and after drying.

### 4.12. Histology

Approximately 0.5 × 1 cm^2^ of full-thickness skin was sampled from the back of each mouse. The skin sample was fixed in 10% neutral buffered formalin before staining (H&E, Masson’s trichrome, resorcin-fuchsin, Sirius red).

### 4.13. Enzyme-Linked Immunosorbent Assay (ELISA)

The concentration of matrix metalloproteinase (MMP)-1 and MMP-3 in the supernatants was determined. Briefly, we took the dorsal skin tissue (0.1 g) of the mice, used 9× saline per tissue weight, homogenized on ice, and undertook centrifugation (5000× *g*, 15 min, room temperature). After collecting the supernatant, we measured the MMP-1 and MMP-3 concentrations according to the instructions in the ELISA kits (Beijing Solarbio Science & Technology, Beijing, China).

### 4.14. Statistical Analyses

The data were processed using Prism 8.0 (GraphPad, La Jolla, CA, USA). The data are presented as the mean ± standard deviation. Differences in the data between two groups were compared using the independent-sample *t*-test. *p* < 0.05 was considered significant.

## 5. Conclusions

Positively charged ACS can form stable-sized homogeneous ANGAs by electrostatic adsorption with negatively charged NGAs. The ANGAs showed no significant change in particle size and cytotoxicity after high-temperature sterilization. The skin-permeation ability of ANGA hydrogel was improved compared to that of NGA hydrogel. Furthermore, the ANGA hydrogel could inhibit the UV-induced expression of MMP-1 and MMP-3, then inhibit ECM degradation, increase the collagen content in the dermis, maintain the elasticity of the skin, and achieve an antagonistic effect upon skin photoaging. The ANGA hydrogel can employ multiple molecular mechanisms to resist photoaging.

## Figures and Tables

**Table 1:** Effect of the ACS: NGA ratio on the preparation of composites (mean ± SD, *n* = 3).

ACS: NGA (*w*/*w*)	Zeta Potential (mV)	Mean Particle Size (nm)	PDI
0	−36.80 ± 0.70	290.6 ± 7.3	0.13 ± 0.06
1:20	14.96 ± 0.53	306.3 ± 4.2	0.19 ± 0.07
1:10	31.63 ± 0.76	314.3 ± 4.5	0.16 ± 0.05
1:5	33.56 ± 0.74	316.0 ± 3.6	0.19 ± 0.06
1:1	36.67 ± 0.72	320.3 ± 6.5	0.18 ± 0.05

**Table 2:** Changes in mean particle size and zeta potential of NGA and ANGA suspensions before and after sterilization (mean ± SD, *n* = 3).

	Before Sterilization		After Sterilization	
	NGA	ANGA	NGA	ANGA
MPS (nm)	290.6 ± 7.3	314.3 ± 4.5	360.2 ± 4.8	318.8 ± 5.4
Zeta potential (mV)	−36.80 ± 0.70	31.63 ± 0.76	−35.45 ± 1.1	30.88 ± 1.4
PDI	0.13 ± 0.06	0.16 ± 0.05	0.16 ± 0.08	0.17 ± 0.09

**Table 3:** Cumulative permeability and percent retention of GA in hydrogel after 24 h (mean ± SD, *n* = 3).

Sample	Retention Rate (%)	Cumulative Permeability (%)
NGA hydrogel	16.9 ± 1.7	56.5 ± 1.4
ANGA hydrogel	14.5 ± 1.4	75.3 ± 1.8

**Table 4:** Assessment of photoaging using grades.

Score	Features Used for Grading
0	No wrinkles or laxity; fine striations running the length of the body
1	Fine striations
2	Disappearance of all fine striations
3	Shallow wrinkles
4	A few deep wrinkles and laxity
5	Increased number of deep wrinkles
6	Severe wrinkles; development of lesions/tumors

## Data Availability

Data are contained within the article.

## Sample Availability

Samples of the compounds are available from the authors upon reasonable request.
